# Prevalence of *BRCA1* and *BRCA2* mutations in non-familial breast cancer patients with high risks in Korea: The Korean Hereditary Breast Cancer (KOHBRA) Study

**DOI:** 10.1007/s10549-012-2001-0

**Published:** 2012-03-02

**Authors:** Byung Ho Son, Sei Hyun Ahn, Sung-Won Kim, Eunyoung Kang, Sue K. Park, Min Hyuk Lee, Woo-Chul Noh, Lee Su Kim, Yongsik Jung, Ku Sang Kim, Dong-Young Noh, Byung-In Moon, Young Jin Suh, Jeong Eon Lee, Doo Ho Choi, Sung Yong Kim, Sung Hoo Jung, Cha Kyong Yom, Hyde Lee, Jung-Hyun Yang, the KOHBRA Research Group and the Korean Breast Cancer Society

**Affiliations:** 1 Department of Surgery, College of Medicine, University of Ulsan and Asan Medical Center, Seoul, Korea; 2 Department of Surgery, Breast and Endocrine Service, Seoul National University Bundang Hospital, Seongnam, Gyeonggi-do Korea; 3 Department of Preventive Medicine, Seoul National University College of Medicine, Cancer Research Institute, Seoul, Korea; 4 Department of Biomedical Science, Seoul National University College of Medicine, Seoul, Korea; 5 Department of Surgery, College of Medicine, Soonchunhyang University, Seoul, Korea; 6 Department of Surgery, Korea Institute of Radiological and Medical Science, Korea Cancer Center Hospital, Seoul, Korea; 7 Division of Breast and Endocrine Surgery, Hallym University Sacred Heart Hospital, Anyang, Korea; 8 Department of Surgery, School of Medicine, Ajou University Hospital, Suwon, Korea; 9 Department of Surgery, Cancer Research Institute, Seoul National University College of Medicine, Seoul, Korea; 10 Department of Surgery, Ewha Womans’ University Hospital, Seoul, Korea; 11 Department of Surgery, St. Vincent’s Hospital, The Catholic University of Korea, School of Medicine, Suwon, Korea; 12 Department of Surgery, Samsung Medical Center, Sungkyunkwan University, Seoul, Korea; 13 Department of Radiation Oncology, Samsung Medical Center, Sungkyunkwan University, Seoul, Korea; 14 Department of Surgery, Soonchunhyang University Hospital, Choenan, Korea; 15 Department of Surgery, Chonbuk National University Hospital, Jeonju, Korea; 16 Department of Surgery, College of Medicine, Yonsei University, Gangnam Serverance Hospital, Seoul, Korea; 17 Department of Surgery, Konkuk University Medical Center, Seoul, Korea

**Keywords:** *BRCA1*, *BRCA2*, Hereditary breast cancer, Korean, Non-familial, Prevalence

## Abstract

Prevalence and phenotype of *BRCA* mutation can vary by race. The purpose of this study is to evaluate the prevalence of *BRCA1/2* mutations in non-familial breast cancer patients with high risks in Korea. A subset of 758 patients was selected for this study from the KOHBRA nationwide multicenter prospective cohort study. Mutations in *BRCA1/2* genes were tested using fluorescent-conformation sensitive gel electrophoresis, denaturing high performance liquid chromatography or direct sequencing. Mutation of *BRCA1/2* genes were identified in 65 (8.6%) patients among total 758 patients [*BRCA1* mutation: 25 (3.3%), *BRCA2* mutation: 40 (5.3%)]. According to risk groups, mutation of *BRCA1/2* genes were identified in 53 (8.5%) of 625 early onset patients (age ≤40), in 22 (17.7%) of 124 bilateral breast cancer patients, in 3 (50.0%) of 6 breast and ovarian cancer patients, in one (5.9%) of 17 male breast cancer patients, in 5 cases (7.6%) of 66 multiple organ cancer patients. The most common mutation was 509C>A for *BRCA1* and 7708C>T for *BRCA2*. The prevalence of *BRCA1/2* mutations by age in early onset patients was significantly different (age <35 vs age ≥35; 10.0 vs 2.9%, *p* = 0.0007). *BRCA1/2* mutations for non-familial Korean breast cancer patients were detected at a high rate, particularly, in patients with early onset of less than 35 years of age, bilateral breast cancer, and breast and ovarian cancer. Individualized genetic counseling should be offered for non-familial breast cancer patients with these risk factors.

## Introduction

Although hereditary breast cancer is responsible for about 5–10% of all breast cancers, women carrying *BRCA1* and *BRCA2* gene mutations have especially high risk of breast cancer development. Mutations in *BRCA1/2* genes have an estimated lifetime risk of breast cancer between 60 and 85%, and a lifetime risk of ovarian cancer between 26 and 54% for *BRCA1* and between 10 and 23% for *BRCA2*. They are responsible for about 45% of families with multiple cases of breast cancer and up to 90% of families with both breast and ovarian cancer [1–4]. There are specific clinical and pathological features associated with hereditary *BRCA1* or *BRCA2* mutation associated breast cancers [5–7].

Genetic counseling and genetic testing to identify *BRCA1* and *BRCA2* gene mutations in high risk (a significant risk ≥10% of mutation) patients is widely available and commonly employed in the US and Europe [1, 8]. Management for breast cancer patients with a *BRCA* mutation should be established based on the clinicopathologic status as well as the result of genetic testing. Both prophylactic mastectomy and prophylactic oophorectomy are performed in approximately one-third and one half of mutation carriers in Western countries, respectively [9, 10]. However, decision-making for specific management should be done by a physician knowledgeable about the implications and lifetime risks of both breast and ovarian cancer.

There are some limitations to applying the clinical practice guidelines for genetic testing based on data from Western countries due to a lack of *BRCA* mutation data in breast cancer patients of Korean and other racial backgrounds [1, 11, 12]. First, the clinicopathological features associated with breast cancer may differ between races. For example, the proportion of triple negative breast cancer is higher in African-Americans and Asians compared to Caucasian Americans [13, 14]. Although the incidence of breast cancer in Asian countries, such as Korea, is lower than in Europe and the US, the incidence of Korean breast cancer has increased continuously by about threefold during the last two decades. The most striking difference compared to that of Western countries is that breast cancer among Korean women develops at a younger age [15, 16]. Sixty percent of patients in Korea are premenopausal compared to 30% in the United States. As ER-negative breast cancers are more common in premenopausal women, one may expect differences in frequencies of molecular subtypes of breast cancer between Korean and Caucasian women [13, 17]. There may be differences between countries in terms of genetic–environment interaction for development of breast cancer, ethnic diet, or environmental exposures. Consideration of family structure between in Korea and in Western countries is also needed because smaller family size makes family history less useful for identification of germline genetic risk [18].

Prevalence and phenotype of *BRCA* mutation varies according to country and race [19–21]. Several reports on *BRCA* gene mutation in Korean breast cancer patients have been published over a decade since a first report of *BRCA1* among Korean pedigrees in 1995 [22–30]. The prevalence of *BRCA1* and *BRCA2* mutations were reported in 2.5–3.1% for sporadic breast cancers, in 19.4–42.9% for familial breast cancer patients with two or more affected first- and second-degree relatives with breast or ovarian cancers, and in 9.6–18.3% for early onset breast cancers. *BRCA1* and *BRCA2* mutations were also identified in 15.4% for bilateral breast cancer and in 17.9% for multiple organ cancer, and *BRCA2* mutations were identified in 25% for male breast cancer in one study [27]. However, the data in high-risk breast cancer patients without family history of breast or ovarian cancer did not accurately reflect the prevalence of *BRCA* mutation in Korean breast cancer patients because of the very small sample size. A large scale multicenter study was needed to evaluate the prevalence of *BRCA* mutation among Korean breast cancer patients nationwide.

The KOHBRA study was designed by the Korean Breast Cancer Society to investigate the prevalence of *BRCA1/2* mutations in several groups of subjects seen in high-risk breast cancer clinics in Korea between 2007 and 2010 [31]. These groups included patients with a family history of breast or ovarian cancers, patients with non-familial breast and ovarian cancer but with other risk factors of genetic disease, and family members of breast cancer patients with *BRCA1/2* mutations. The KOHBRA study also investigated the prevalence of ovarian cancer in females with *BRCA1/2* mutations

The present study evaluated the prevalence of *BRCA1* and *BRCA2* mutations in the second KOHBRA group, breast cancer patients without family history of breast or ovarian cancer with other risk factors for genetic predisposition.

## Methods

### Patients

The nationwide prospective cohort “KOHBRA” study accrued 1,967 subjects between May 2007 and May 2010 from 35 hospitals registered in the Korean Breast Cancer Society. The overall protocol is described more in detail in the interim report of the KOHBRA study [31, 32]. Inclusion criteria for this substudy are: patients without family history of breast or ovarian cancer but with at least one of the following risk factors for hereditary predisposition; early onset breast cancer defined as diagnosis at or younger than age 40, bilateral breast cancer, personal history of breast and ovarian cancer, male breast cancer, or cancer of multiple organs that include breast. This study received IRB approval from the individual institutes at the beginning of the study and consent from the study patients at the time of enrollment. Patients who were unable to communication or who did not return the questionnaire were excluded. Seven hundred and fifty-eight patients from the KOHBRA cohort met the criteria for this study.

Among the 758 patients, 741 (97.8%) patients were female and 17 (2.2%) patients were male. The breakdown of the high-risk groups is shown in Table 1. There were 550 (72.6%) patients with early onset breast cancer, 67 (8.8%) patients with bilateral breast cancer, 42 (5.5%) patients with cancer of multiple organs, 15 (2.0%) male breast cancer patients, 5 (0.7%) patients with both breast and ovarian cancer, and 79 (10.4%) patients having two or more of these high genetic risk factors.

**Table 1:** Classification of high-risk groups

High-risk classification	No of patients (%)
Early onset breast cancer (age ≤40 years) (E)	550 (72.6)
Bilateral breast cancer (B)	67 (8.8)
Male breast cancer (M)	15 (2)
Breast and ovarian cancer (BO)	5 (0.7)
Multiple organ cancer patients including breast (Mu)	42 (5.5)
Two or more of these high risks^a^	79 (10.4)
Total	758 (100.0)

^a^ E + B:53, E + M:1, E + BO:1, E + Mu:19, B + Mu:3, M + Mu:1, E + B + Mu:1

### Collecting clinical data

The family history and epidemiological data was obtained by a baseline questionnaire, the anthropometric data was measured, and the clinical information was collected by physician chart-reviews. Questionnaire included information about basic clinical data, past history, familial cancer history, life style, reproductive factors, social-psychological factors, and history of complementary therapies, etc. Physical information like body weight, height, body circumference, and body composition was acquired via physical examination at the same time of questionnaire. Pedigree of at least three generations was acquired via genetic counseling in all patients. All the data was computerized and regularly reviewed through quality control for missing or error data. Follow-up data of a new cancer, recurrence, and mortality was collected at 1 and 3 years after enrollment.

### *BRCA1* and *BRCA2* genomic mutation analysis

Genomic DNA was extracted from the patients’ peripheral blood. Mutations in *BRCA1* and *BRCA2* were scanned through the 22 coding exons of *BRCA1* and 26 coding exons of *BRCA2* using fluorescence-conformation sensitive gel electrophoresis (F-CSGE) or denaturing high performance liquid chromatography (DHPLC). Following identification of a subset of PCR products with aberrant patterns, direct sequencing was performed in these DNA fragments on an ABI3100 or ABI3700 (Applied Biosystems, CA) or a MegaBACE500 (GE Healthcare, UK) genetic analyzer, following the manufacturer’s instructions. Scanning and direct sequencing of *BRCA* genes was performed based on the institutes’ own protocol and equipment. Some institutes performed direct sequencing through the entire *BRCA1* and *BRCA2* genes. Among all 758 patients, an F-CSGE performed in 670 cases (88.4%), DHPLC in 14 cases (1.8%), and direct sequencing in 74 cases (9.8%). In this study, the definition of a genetic mutation is confined to the protein-truncating mutation and the missense mutation, which have a confirmed association to disease. Unclassified variants were not considered as a genetic mutation.

## Results

### Prevalence of *BRCA1* and *BRCA2* mutations in high-risk breast cancer patients without family history of breast or ovarian cancer

**Fig. 1:**
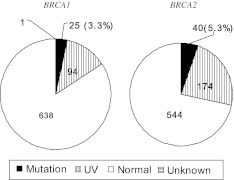
Prevalence of *BRCA1* and *BRCA2* mutations in high-risk breast cancer patients without family history of breast or ovarian cancer. Overall *BRCA1/2* mutations: 65^†^/758 (8.6%), ^†^One patient had both *BRCA1* and *BRCA2* mutations. *UV* unclassified variant

Overall, mutation of *BRCA1* or *BRCA2* genes was identified in 65 (8.6%) patients among 758 high-risk breast cancer patients without family history of breast or ovarian cancer. *BRCA1* mutation was found in 25 (3.3%) patients and *BRCA2* mutation was found in 40 (5.3%) patients. One patient had both *BRCA1* and *BRCA2* mutations (Fig. 1).

According to each high-risk group, mutation of *BRCA1* and *BRCA2* genes were identified in 53 (8.5%) of 625 early onset patients, in 22 (17.7%) of 124 patients with bilateral breast cancer, in 3 (50.0%) of 6 patients with breast and ovarian cancer, in 1 (5.9%) of 17 male breast cancer patients, in 5 (7.6%) of 66 patients with cancer of multiple organs including breast, and in 19 (27.1%) of 79 patients having two or more of these high risks (Table 2). Using non-overlapping risk groups, mutation of *BRCA1* or *BRCA2* genes were identified in 35 (6.4%) of 550 early onset patients, in 5 (7.5%) of 67 patients with bilateral breast cancer, in 2 (40.0%) of 5 patients with breast and ovarian cancer, in 1 (6.7%) of 15 male breast cancer patients, and in 3 (7.0%) of 42 patients with multiple organ cancers including breast.

**Table 2:** *BRCA1* and *BRCA2* mutations according to high-risk groups

*BRCA* mutation	Early onset ≤40 yrs	Bilateral breast cancer	Male breast cancer	Breast and ovarian cancer	Multiple organ cancer	Two or more high risks
*BRCA1/2*						
Mutation no. (%)	53 (8.5)	22 (17.7)	1 (5.9)	3 (50.0)	5^b^ (7.6)	19 (27.1)
No mutation no. (%)	572 (91.5)	102 (82.3)	16 (94.1)	3 (50.0)	62 (92.4)	60 (75.9)
*BRCA1* ^c^						
Mutation no. (%)	22 (3.5)	7 (5.6)	0 (0.0)	1 (16.7)	2 (3.0)	7 (8.9)
No mutation no. (%)	523 (83.7)	97 (78.2)	17 (100.0)	5 (83.3)	58 (87.9)	62 (78.5)
UV^a^ no. (%)	79 (12.6)	19 (15.3)	0 (0.0)	0 (0.0)	6 (9.1)	9 (11.4)
*BRCA2*						
Mutation no. (%)	31 (5.0)	15 (12.1)	1 (5.9)	2 (33.3)	3 (4.6)	12 (15.2)
No mutation no. (%)	456 (73.0)	85 (68.5)	12 (70.6)	3 (50.0)	42 (63.6)	53 (67.1)
UV^a^ no. (%)	138 (22.1)	24 (19.4)	4 (23.5)	1 (16.7)	21 (31.8)	14 (17.7)
Total	625	124	17	6	66	79

^a^ *UV* unverified mutation

^b^One patient had both *BRCA1* and *BRCA2* mutations

^c^Data of *BRCA1* test in one patient was not available

### *BRCA1* and *BRCA2* mutation by risk groups

*BRCA1* mutations were detected in 22 (3.5%) of 625 early onset patients, in 7 (5.6%) of 124 patients with bilateral breast cancer, in 1 (16.7%) of 6 patients with breast and ovarian cancers, in 2 (3.0%) of 66 patients with multiple organ cancers, and in 7 (8.9%) of 79 patients having two or more of these high-risk factors. There was no BRCA1 mutation detected among 17 male breast cancer patients.

*BRCA2* mutations were detected in 31 (5.0%) early onset patients, 15 (12.1%) patients with bilateral breast cancer, 2 (33.3%) patient with breast and ovarian cancer, 3 (4.6%) patients with multiple organ cancers, 1 (5.9%) male breast cancer patient, and 12 (15.2%) patients having two or more of these high risks (Table 2).

### *BRCA1* and *BRCA2* mutations in early onset breast cancer patients

Patients with early onset breast cancer had a variety of *BRCA1* and *BRCA2* mutations based on the combined risk groups. Of 625 early onset patients, *BRCA1* and *BRCA2* mutations were detected in 35 (6.4%) of 550 patients without other risks, in 16 (30.2%) of 53 patients with bilateral breast cancer, in one (100%) patient who also had ovarian cancer, and in one (5.3%) of 19 patients with multiple organ cancers (Table 3).

**Table 3:** *BRCA1* and *BRCA2* mutations in patients with early onset breast cancer diagnosed at ≤40 years

Risk classification	<35 years		≥35 years		Total	
	Patients no.	Mutation no. (%)	Patients no.	Mutation no. (%)	Patients no.	Mutation no. (%)
Early onset breast cancer ≤40 yrs only (E)^*^	271	27 (10)	279	8 (2.9)	550	35 (6.4)
E + Bilateral breast cancer	20	7 (35)	33	9 (27.3)	53	16 (30.2)
E + Breast and ovarian cancer	0	0	1	1 (100)	1	1 (100)
E + Multiple organ cancer	5	0	14	1 (7.1)	19	1 (5.3)
E + Male breast cancer	1	0	0	0	1	0
E + Bilateral breast cancer + multiple organ cancer	1	0	0	0	1	0
Total	298	34 (11.4)	327	19 (5.8)	625	53 (8.5)

* *p* = 0.0007

According to age at diagnosis, *BRCA1* and *BRCA2* mutations were detected in 24.0% of patients 40 years or younger with other risk factors and in 6.4% among early onset patients without other risk factors. However, among the early onset patients without other risk factors, *BRCA1 and BRCA2* mutations were detected in 10.0% in patients less than 35 years and in 2.9% in patients 35 years or older (*p* = 0.0007) (Table 3).

In terms of age group, incidence of *BRCA1/2* mutations in patients 35 years or older showed a significant difference depending on whether other risks were combined or not (22.9 vs 2.9%, *p* = <0.0001). On the other hand, *BRCA1/2* mutations in patients less than 35 years showed high incidence rates regardless of the combined risks (25.9 vs 10%, *p* = 0.01) (Table 4).

**Table 4:** *BRCA1/2* and *BRCA2* mutations by age in patients with early onset breast cancer diagnosed at ≤40 years

Age	Risk	*BRCA1/2*		Total no. (%)	*p* value
		Mutation	No mutation		
≥35 yrs	With other risks^a^	11	37	48	<0.0001
	No. (%)	22.9	77.1	14.7	
	Without other risks	8	271	279	
	No. (%)	2.9	97.1	85.3	
	Total	19	308	327	
	No. (%)	5.8	94.2	100	
<35 yrs	With other risks	7	20	27	0.01
	No. (%)	25.9	74.1	9.1	
	Without other risks	27	243	270	
	No. (%)	10	90	90.9	
	Total	34	263	298	
	No. (%)	11.4	88.6	100	

^a^Other risks included early onset, bilateral breast cancer, male breast cancer, breast and ovarian cancer, and multiple organ cancer

We evaluated each patient’s family history to develop a family structure variable to find whether a limited family structure affects *BRCA1/2* mutation prevalence or not. Limited family structure was defined as fewer than two first- or second-degree female relatives surviving past age 45 years in either the maternal or paternal lineage. Otherwise the patient was deemed to have an adequate family structure [18]. *BRCA1* and *BRCA2* mutations did not show different incidence between limited structure and adequate structure according to family structure in early onset patients (Table 5).

**Table 5:** *BRCA1/2* and *BRCA2* mutations according to family structure in patients with early onset breast cancer diagnosed at ≤40 years

Age		Family structure		*p* value
		Limited (*n* = 146)	Adequate (*n* = 92)	
*BRCA1/2*				
Age <35 yrs				
Mutation no. (%)		16 11.0	9 9.8	0.77
No mutation no. (%)		130 89.0	83 90.2	
Age ≥35 yrs		Limited (*n *= 186)	Adequate (*n* = 90)	
Mutation no. (%)		4 2.7	1 1.1	0.4
No mutation no. (%)		142 97.2	89 98.9	
All		Limited (*n *= 292)	Adequate (*n* = 182)	
Mutation no. (%)		20 6.9	10 5.5	0.56
No mutation no. (%)		272 93.1	172 94.5	

### Possible candidates for founder mutation of *BRCA1* and *BRCA2* genes

Overall 43 mutations (18 *BRCA1* genes and 25 *BRCA2* genes) were identified in high-risk breast cancer patients without a family history of breast or ovarian cancer. The most common *BRCA1* mutation gene was 509C>A, which was identified separately in four unrelated patients. 1041delAGCinsT, 1630insG, 3746insA, and 5615del11insA were identified separately in two unrelated patients. These five *BRCA1* genes accounted for 48% of the *BRCA1* mutations identified. The most common *BRCA2* mutation gene was 7708C>T, which was identified separately in six unrelated patients. 584delTTAA and 6952delGA was identified in three unrelated patients. 2041dupA, 8542G>T, 1672A>T, 9219T>G, 9481delA, and 999del5 were separately identified in two unrelated patients. These nine *BRCA2* genes accounted for 60% of the *BRCA2* mutations identified (Table 6).

**Table 6:** Frequency of *BRCA1* and *BRCA2* mutations in high-risk breast cancer patients without family history of breast or ovarian cancer

	BIC nomenclature	Effect on amino acid	*n* ^a^	%
BRCA2	7708C>T	p.Arg2494X hetero	6	9.2
BRCA1	509C>A	p.Tyr130X hereto	4	6.2
BRCA2	5804delTTAA	p.I1859KfsX3 hetero	3	4.6
BRCA2	6952delGA^b^	p.Asp2242PhefsX2 hetero	3	4.6
BRCA1	1041delAGCinsT	p.Ser308X hetero	2	3.1
BRCA1	3746insA	p.Glu1210ArgfsX9 hetero	2	3.1
BRCA1	5615del11insA^c^	p.Val1833SerfsX7 hetero	2	3.1
BRCA1	1630dupG	p.Lys505X hetero	2	3.1
BRCA2	1627A>T	p.Lys467X hetero	2	3.1
BRCA2	2041dupA	p.I605NfsX11 hetero	2	3.1
BRCA2	8542G>T^b^	p.Glu2772X hetero	2	3.1
BRCA2	9219T>G^b^	p.Tyr2997X hetero	2	3.1
BRCA2	9481delA	p.Thr3085GlnfsX19 hetero	2	3.1
BRCA2	999del5	p.Asn257LysfsX17 hetero	2	3.1

^a^Frequency in disease-causing mutation (*n* = 65)

^b^Novel

^c^Detected in only Korean

## Discussion

Overall mutation of *BRCA1* and *BRCA2* genes in high-risk breast cancer patients without family history of breast or ovarian cancer in this study was 8.6% (*BRCA1*: 3.3%, *BRCA2*: 5.3%). The prevalence of genetic BRCA1/2 mutation among non-familial patients with high-risk factors in this study was more than three times higher than the 2.6% prevalence observed for *BRCA1*/2 mutations in sporadic breast cancer patients in Korea [25, 27, 28]. Genetic counseling and testing for *BRCA1* and *BRCA2* gene mutation has become an integral part of high-risk patient evaluation and management for hereditary breast ovarian cancer [11, 33]. Guideline organizations, including NCCN and ASCO, have advised targeting *BRCA1* and *BRCA2* testing to probands whose probability of mutation carriage is approximately 10% or greater. However, there might be variation in *BRCA1/2* mutation prevalence across race and ethnicity for breast cancer patients with high-risk factors, although the prevalence of *BRCA1/2* mutations is comparable among sporadic breast cancer patients of African, Asian, White, and Hispanic descent: approximately 1–4% per gene, with the exception of Ashkenazi Jewish and Icelandic patients [33, 34]. There is a question of whether ethnic differences in genetic, reproductive, or environmental exposures might alter mutation penetrance, which could have significant implications for clinical practice. Therefore, large data collection of *BRCA1/2* mutation prevalence and penetrance in individual ethnic groups is necessary for applying individualized guideline for genetic counseling and testing, and management in clinical practice.

This KOHBRA study showed the prevalence of *BRCA1/2* mutations in high-risk breast cancer patients without family history of breast or ovarian cancer via a large multicenter nationwide cohort study. Previous reports of *BRCA1/2* mutations in Korean breast cancer patients were limited by small sample sizes, and the studies were performed individually at various sites with different screening methods. Overall mutation of *BRCA1/2* genes in high-risk breast cancer patients without family history of breast or ovarian cancer in this study was 8.6%. On the other hand, the prevalence of *BRCA1/2* mutations among breast cancer patients with family history of breast or ovarian cancers was 24.8% in the KOHBRA study interim report [32]. Notably, *BRCA2* mutation was more common than *BRCA1* mutation in our study, compared with previous small clinic-based studies [25–27]. This finding is consistent with other studies showing *BRCA2* mutations appear as or more commonly than *BRCA1* mutations in clinic-based Asians, a difference from other ethnic groups [34].

According to high-risk groups in this study, mutation of *BRCA1/2* genes were identified in 8.5% among early onset patients, in 17.7% among bilateral breast cancer, in 50.0% among patients with both breast and ovarian cancer, in 5.9% among male breast cancer patients, in 7.6% among patients with cancer of multiple organs, and in 27.1% among patients having two or more of these high-risk factors. There are few previous smaller studies of Korean patients which report *BRCA* mutations by individual risk category and several of these include patients with family history [24, 27]. *BRCA1/2* mutations in Korean patients with early onset regardless of family history were detected in 10.4–18.3% in the previous studies (Table 7) [23, 26, 32]. The wide variation in reported prevalence of *BRCA1/2* mutation in patients with early onset could be related to variation in number with family history of breast cancer in these studies. When the group of early onset patients under 35 years was considered, we found prevalence of 10% for *BRCA1/2* mutations with no other risk factor. The mutation rate increased up to 25.9% for the early onset patients with other risk factor. Therefore, additional risk factors increase the likelihood of BRCA1/2 mutation among young patients.

**Table 7:** Prevalence of *BRCA1* and *BRCA2* mutations among early onset breast cancer patients with or without familial history of breast or ovarian cancers in Korea

	Ahn et al. [26]	Kang et al. [23]	KOHBRA study-interim report [32]
No. of cases tested (Age)	183 (Age <35)	60 (Age <40)	513 (Age ≤40)
History of familial breast or ovarian cancers (%)	24 (13.1)	8 (22.0)	181 (32.0)
*BRCA1* mutation (%)	13 (7.1)	6 (10.0)	48 (9.4)
*BRCA2* mutation (%)	6 (3.3)	5 (8.3)	43 (8.4)
*BRCA1/2* mutations (%)	19 (10.4)	11 (18.3)	91^a^ (17.7)

^a^Two cases of both *BRCA1* and *BRCA2* mutations

Two studies reviewed the prevalence of *BRCA1/2* mutations in breast cancer patients in terms of ethnic difference, finding that those diagnosed at a younger age (mostly under 35 to 45-year old) had prevalence of 5–10% among various races [24, 34]. On the other hand, the prevalence of *BRCA1* mutation was particularly high in African-American patients diagnosed before age 35 years (16.7%), compared with young Hispanics (8.9%), non-white Hispanics without Ashkenazi Jewish ancestry (7.2%), and Asian-Americans (2.4%) [35].

Family structure can affect *BRCA* gene mutation rate and the accuracy of mutation probability models. *BRCA1/2* mutations were detected in 13.7% of the limited family structure and in 5.2% of the adequate family structure. Family structure was a significant predictor of mutation status [18]. However, there was no significant difference of *BRCA* gene mutations between family structures in our study.

The prevalence of *BRCA1/2* mutations in bilateral breast cancer patients in Korea was 17.7% in our study and was higher than 15.4% prevalence in a previous small study [27]. *BRCA1/2* mutations of bilateral breast cancer in other ethnic countries showed both 29.6% in high-risk Jewish families and in Polish patients with having 46.9–82.4% positive familial history [36, 37].

There is a lack of studies about the prevalence of *BRCA1/2* mutations in male breast cancer in Korea. Two (25%) *BRCA2* mutations and no *BRCA1* mutation were reported among eight male breast cancer patients in a small study [27]. However, the patients with mutation also had familial breast cancer history. Our study had a small number of male breast cancer patients and *BRCA2* mutation was detected in only one (5.9%) patients. The *BRCA* mutations in male breast cancer without familial breast cancer history are usually undetected and if detected, it is almost always in *BRCA2* gene.

The prevalence of *BRCA2* mutation in male breast cancer varies from 4 to 40% according to racial differences. This variance might be related to the different rates of familial breast cancer history [38–43]. On the other hand, a recently published study accounted for 16% *BRCA2* mutation of 115 male breast cancer cases from the United States, including 40% for breast cancer families and 13% for non-familial breast cancer. The study suggested that family history is not a strong predictor of carrying a mutation in males and males who develop breast cancer should be screened for mutations in *BRCA2* [44].

The prevalence of *BRCA1/2* in multiple organ cancer cases in our study (7.6%) was lower than previously reported (9.1%) for cases without familial history in a previous small study, which also showed 50% with *BRCA1/2* mutations in cases with familial breast cancer history [27]. In our study, the other primary cancer sites in breast cancer patients were 46 thyroid cancers, 6 uterine cancers, 5 kidney cancers, 2 stomach cancers, 2 rectal cancers, 2 tongue cancers, 1 liver cancer, 1 pancreatic cancer, and 1 bone cancer. Five *BRCA1/2* mutations were detected in 2 thyroid cancers, 2 kidney cancers, and 1 bone cancer, respectively (Table 8). It would seem that genetic testing should be considered whether or not a specific primary cancer is genetically related to breast cancer. For example, the International Consensus Conference on Breast Cancer Risk, Genetics, and Risk Management suggested as candidates for genetic counseling and testing for breast cancer, any patient who has had a family history of prostate cancer, thyroid cancer, sarcoma, endometrial cancer, adrenocortical cancer, brain cancer, or pancreatic cancer—although these involve less strict criteria [1].

**Table 8:** *BRCA1* and *BRCA2* mutations in patients with multiple organ cancer

	Number of total subjects	BRCA1 mutation no.	BRCA2 mutation no.	Overall prevalence (%)
Breast and other cancers	66	2	3	7.6
Thyroid cancer	46	1	1	4.3
Uterine cancer	6	0	0	0.0
Kidney cancer	5	1^a^	1^a^	40.0
Stomach cancer	2	0	0	0.0
Rectal cancer	2	0	0	0.0
Tongue cancer	2	0	0	0.0
Liver cancer	1	0	0	0.0
Pancreas cancer	1	0	0	0.0
Bone cancer	1	0	1	100.0

^a^One patient with kidney cancer had both *BRCA1* and *BRCA2* mutations

A study reported that the germline mutation rate in *BRCA1* was 2.7% in 37 Korean sporadic ovarian cancer patients unselected for family history, which was slightly lower than rates obtained in other countries [45]. The prevalence of *BRCA1/2* mutations in patients with both breast and ovarian cancer in our study was 50% and higher than that in Myriad data (20%), although the number of cases were small [46]. This high prevalence supports current practice guidelines of genetic testing for all patients with breast and ovarian cancers, regardless of age or family cancer history [11].

Although germline mutations were scattered through the *BRCA1* and *BRCA2* genes, the *BRCA1*, 509C>A gene which was identified separately in four unrelated patients, and the *BRCA2*, 7708C>T gene which was identified separately in six unrelated patients, are the most frequent mutation in either genes and accounted for 16 and 15% of mutations detected in *BRCA1* and *BRCA2* respectively in this study of Korean women. 509C>A gene was also detected in Asian population and 5615del11insA were detected in only Korean patients in the BIC database. The *BRCA2*, 7708C>T gene was detected in Asian and West European population and detected frequently in Korean patients in the previous studies [23–25, 27–29]. These genes would be possible candidates for founder mutations of *BRCA1/2* in high-risk Korean breast cancer patients. However, defining founder genes in Korean breast cancer patients should be approached carefully, because there is no data of mutation frequency in the Korean general population and additional data about *BRCA* mutations is required even though the KOHBRA study was a nationwide multicenter study.

The selection of genetic testing criteria would be clinically important because the mutation result might affect the decision-making for operation method and follow-up method. Although widely accepted clinical criteria for referral are usually suggested, genetic testing criteria may differ between countries based on mutation prevalence because the prevalence of *BRCA1/2* mutations varies considerably among ethnic groups and geographical areas [12]. As the NCCN guideline currently recommends genetic counseling and genetic testing for any patient with high risk 10% or more of mutation, the prevalence of *BRCA1/2* mutations in high-risk breast cancer patients without family history of breast or ovarian cancer—noted in this study—may play a key role for genetic testing criteria in Korean breast cancer patients with high risk of mutation. *BRCA*PRO and Myriad II, two of the most commonly used computer predictive models on clinical expertise, show accuracy of approximately 80% for whites with strong personal and/or familial history of breast, ovarian, and/or male breast cancer. However since these models show underestimation of Asian mutation carriers because of their differences in performance by race/ethnicity, using these models to predict the *BRCA1/2* mutation probability among Korean patients may result in inaccuracies [34]. Therefore, large nationwide trials like the KOHBRA study are needed to continue developing the appropriate guidelines for genetic counseling, genetic testing, and management as well as the suitable predictive model for *BRCA1/2* mutation patients or carriers in Korea. It is important to evaluate whether the difference of *BRCA1/2* mutation prevalence exists between Koreans and other races. Identifying racial differences in genetic or lifestyle factors, which may modify the cancer risk of *BRCA1/2* mutations, is also a priority for future research. So far, the management for *BRCA1/2* mutation patients or carriers in Korea is recommending that application is individually based on the prevalence data of *BRCA1/2* mutation of the KOHBRA study, as well as the worldwide guidelines such as the NCCN guideline or the International Consensus Conference guideline.

Our study has some limitations despite being a large multicenter nationwide study. Three different screening methods for testing *BRCA1/2* mutations were performed although most patients were tested with the F-CSGE method. This heterogeneous screening method might affect the prevalence of *BRCA* mutation. Also, the number of patients varied according to risk groups. For instance, the prevalence of *BRCA1/2* mutations in male breast cancer patients as well as in patients with breast and ovarian cancer in this study must be interpreted with caution due to the very small number of subjects. Another limitation involves the various additional primary organ sites for patients with multiple organ cancers. Additional primary cancer sites in breast cancer patients in our study were thyroid, uterus, kidney, stomach, tongue, rectum, liver, bone, and pancreas. Whether cancers from these organs are genetically related to breast cancer or BRCA1/2 mutation requires confirmation. Besides the differences in number of cases with familial breast cancer history, the different sites of multiple-organ cancers might affect the many discrepancies in the prevalence of *BRCA1/2* in multiple organ cancers cases between our study and the previous smaller study [27].

In conclusion, *BRCA1* and *BRCA2* mutations were detected at higher frequency than reported for Korean sporadic breast cancer patients in those patients with no family history of breast or ovarian cancer but with other risk factors of genetic disease. In particular, the patients with early onset of less than 35 years of age, bilateral breast cancer, and personal history of breast and ovarian cancer had greater than 10% prevalence of *BRCA1/2* mutations. Genetic testing may be indicated for these high-risk patient groups.
